# Trends in age and prostate-specific antigen at prostate cancer diagnosis between 2010 and 2019

**DOI:** 10.1093/jncics/pkae106

**Published:** 2024-10-23

**Authors:** Lukas Owens, Ojas Brahme, Roman Gulati, Ruth Etzioni

**Affiliations:** Division of Public Health Sciences, Fred Hutchinson Cancer Center, Seattle, WA, USA; Division of Public Health Sciences, Fred Hutchinson Cancer Center, Seattle, WA, USA; Division of Public Health Sciences, Fred Hutchinson Cancer Center, Seattle, WA, USA; Division of Public Health Sciences, Fred Hutchinson Cancer Center, Seattle, WA, USA

## Abstract

Recent studies have shown that de novo metastatic prostate cancer incidence in the United States increased from 2010 to 2019. Plausible explanations include delayed detection after recommendations against prostate cancer screening or upstaging associated with use of more sensitive imaging technologies. Using Surveillance, Epidemiology, and End Results patient cases and controlling for aging of the population, we found the median age and prostate-specific antigen (PSA) level at prostate cancer diagnosis increased by 1.4 years of age (95% CI = 1.3 to 1.5 years) and 1.4 ng/mL (95% CI = 1.4 to 1.5 ng/mL) over this period, consistent with the delayed detection hypothesis. Racial differences were noted, with 75th percentiles of PSA at diagnosis increasing by 4.3 ng/mL (95% CI = 3.7 to 4.8 ng/mL) over this time period for non-Hispanic Black men compared with 3.0 ng/mL (95% CI = 2.8 to 3.2 ng/mL) for non-Hispanic White men. Overall, patient characteristics at diagnosis suggest that delayed detection contributed at least in part to increases in de novo metastatic disease.

Incidence of de novo metastatic prostate cancer in the United States increased during the 2010s, with one estimate showing an increase of 4.5% annually in regional and distant-stage diagnoses between 2014 and 2019.^1^ Additionally, incidence of de novo metastatic disease differs by race and ethnic origin, with Black men showing a greater incidence of de novo metastatic disease in all years but smaller increases over time than non-Black men.^2^^,^^3^ It is unclear whether these trends are attributable to decreased prostate-specific antigen (PSA) screening after changes in screening guidelines or to other factors, such as the increased utilization of advanced imaging technologies at initial staging.

Despite its widespread use since the 1980s, recommendations concerning the use of PSA screening have changed over time. In 2008, the US Preventive Services Task Force (USPSTF) issued a “D” (ie, do not use) recommendation for PSA screening among men aged 75 and older^4^ and, in 2012, extended the “D” recommendation to all ages.^5^ Screening rates decreased after these recommendations.^6^^,^^7^ In 2018, owing in part to increased uptake of conservative management in men diagnosed with low-risk disease, the USPSTF revised to a “C” (ie, informed and shared decision-making between men and their providers) recommendation for men ages 55 to 69,^8^ but whether decreased screening contributed to increased incidence of de novo metastatic disease during the 2010s remains unclear.^9^

At the same time, more advanced imaging technologies and surgical techniques have been implemented in the diagnosis and staging of prostate cancer. These advances could provide another possible explanation for increasing incidence of de novo metastatic diagnoses.

To better understand the rise in de novo metastatic cases in the United States, we examined age and PSA level at diagnosis among prostate cancer patient cases in registry data. If the increasing trend was due to delayed detection, both age and PSA level at diagnosis would increase with calendar year. However, these characteristics would be unaffected by upstaging due to advanced imaging and surgical methods. Further, screening rates differed across self-identified race and ethnic subgroups both before and after the change in recommendations, so we examined trends in age and PSA level at diagnosis within these subgroups.

We analyzed prostate cancer patient cases in the Surveillance, Epidemiology, and End Results (SEER) database.^10^ A case listing of all prostate cancer patient cases diagnosed from 2010 to 2019 was extracted from the SEER-17 database (N = 508 899), which covers approximately 26% of the US population.^11^ Age at diagnosis (median: 66 years; IQR = 60-72) and PSA level at diagnosis (median: 7.0 ng/mL; IQR = 5.0-11.7) were extracted. In this database, age greater than 85 years was censored, and PSA values greater than 98.0 ng/mL were censored. A variable reflecting self-identified race and ethnic origin was also extracted for each patient, with categories non-Hispanic White (68% of cases), non-Hispanic Black (15%), Hispanic (10%), non-Hispanic Asian or Pacific Islander (5%), and non-Hispanic American Indian or Alaska Native (<1%). Patients with unknown PSA level at diagnosis (16% of cases) or unknown race (3% of cases) were excluded. After exclusions, 416 732 cases were included in the analysis. We confirmed that rates of missing data did not vary substantially by year or age. Statistical analysis was conducted using R version 4.3.0. Two-sided *P* values less than .05 are considered statistically significant.

The joint distribution of age and PSA level at diagnosis is visualized for each year in Figure 1, along with the modes of the smoothed distributions, which were used to capture the central tendencies of these distributions while accounting for their censoring. These plots show that, over the period from 2010 to 2019, cases shifted toward older ages and higher PSA levels at diagnosis. The mode age at diagnosis increased by 3.4 years and the mode PSA level at diagnosis increased by 0.4 ng/mL over this period. However, these plots do not take into account demographic changes in the population at risk of developing prostate cancer, which has shifted to older ages over the time period of interest, with the mean age of US men aged 50 to 80 years (a range covering 92% of prostate cancer diagnoses in our data) increasing by 1.3 years from 61.3 years in 2010 to 62.6 years in 2019.^10^

**Figure 1.**
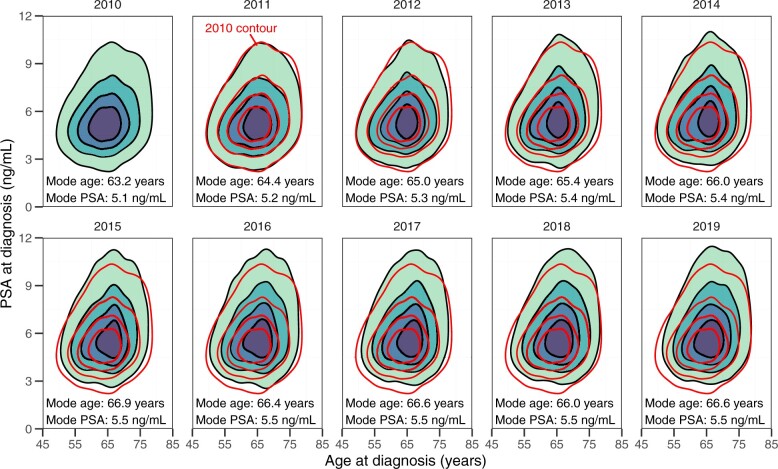
Contour plots and modes of the joint distribution of age and PSA at diagnosis of prostate cancer in the Surveillance, Epidemiology, and End Results database from 2010 to 2019; darker regions indicate higher counts of cases. The 2010 contours are replicated in 2011 to 2019 plots for comparison. Abbreviation: PSA = prostate-specific antigen.

To more formally evaluate changes in the distributions of age and PSA level at diagnosis, we analyzed trends in these characteristics over the calendar year using quantile regressions at the 25th, 50th, 75th, and 85th percentiles. To account for demographic changes, the regressions were weighted by the all-race age distribution of US men in 2010^10^ to effectively hold the population age distribution constant across calendar years. We fit two versions of each model, one that included year as the only predictor and one that also included race and an interaction between year and race. Figure 2 shows the observed quantiles in each year and the fitted regression lines over time for each race group. Tables S1 and S2 detail the fitted regressions of age at diagnosis and PSA level at diagnosis, respectively.

**Figure 2.**
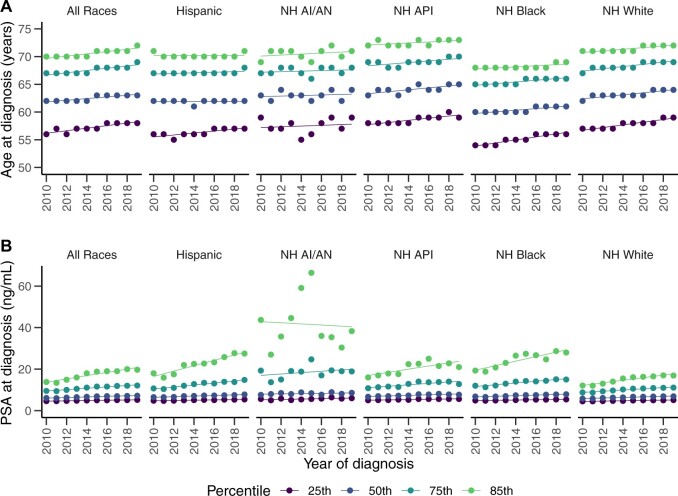
Observed and fitted quantiles of (A) age at diagnosis and (B) PSA at diagnosis by year of diagnosis and race and ethnic origin in the Surveillance, Epidemiology, and End Results database from 2010 to 2019. Abbreviations: PSA = prostate-specific antigen; NH = non-Hispanic; API = Asian/Pacific Islander; AI/AN = American Indian/Alaska Native.

All quantiles studied showed statistically significant increases over time for both outcomes. The median age at diagnosis increased by 0.14 years (95% CI = 0.13 to 0.15 years) annually, for an overall increase of 1.4 years over the entire period, and similar trends were seen in the other quantiles, suggesting a uniform shift of the distribution of age at diagnosis. Median PSA level at diagnosis increased by 0.14 ng/mL (95% CI = 0.14 to 0.15 ng/mL) annually, for an overall increase of 1.4 ng/mL over the period. Greater increases were seen in the 75th and 85th percentiles, which increased by 0.34 ng/mL (95% CI = 0.32 to 0.35 ng/mL) and 0.80 ng/mL (95% CI = 0.78 to 0.85 ng/mL) annually, respectively. These increases in the outer percentiles indicate that the distributions of PSA level at diagnosis tended to become more skewed over time, creating heavier upper tails.

Statistically significant differences in the trends for certain percentiles of PSA level at diagnosis were observed between the race and ethnic origin groups. Trends in the 25th and 50th percentiles for PSA levels did not show statistically significant differences by race, but the non-Hispanic Black and Hispanic groups showed higher trends in the 75th and 85th percentiles. Non-Hispanic Black men showed annual increases of 0.43 ng/mL (95% CI = 0.37 to 0.48 ng/mL) and 1.11 ng/mL (95% CI = 0.90 to 1.32 ng/mL) and Hispanic men showed annual increases of 0.49 ng/mL (95% CI = 0.42 to 0.56 ng/mL) and 1.38 ng/mL (95% CI = 1.14 to 1.60 ng/mL) in the 75th and 85th percentiles of PSA levels, respectively, whereas for non-Hispanic White men, the corresponding annual increases were 0.30 ng/mL (95% CI = 0.28 to 0.32 ng/mL) and 0.65 ng/mL (95% CI = 0.61 to 0.69 ng/mL). Inference for the non-Hispanic Asian or Pacific Islander and non-Hispanic American Indian or Alaska Native groups was unreliable due to the small numbers of these cases.

Overall trends toward older ages and higher PSA levels at diagnosis give support to the hypothesis that delayed detection contributed to increased de novo metastatic prostate cancer incidence. Broad consistency across race and ethnic subgroups is also generally supportive of a role for delayed diagnosis, and differences in results by race and ethnic subgroups may reflect differing trends in PSA test usage. Our general finding does not rule out simultaneous stage inflation due to increased use of more sensitive imaging, nor does it rule out unobserved changes in underlying biological cancer risk or other relevant factors. However, this study does show that changes in patient characteristics at diagnosis are consistent with a delay in detection. This finding is complementary to previous studies that linked increasing de novo metastatic disease to decreased screening in other ways—for example, with a model-based approach.^9^

This study focuses on characteristics of prostate cancer patients available before diagnosis and did not examine trends in features assessed as part of or after diagnostic workup, such as tumor stage or grade, although previous studies have examined this question.^12^ Geographic region is another feature observable before diagnosis, and work in this area is maturing^13^^,^^14^ but requires care to properly analyze alongside race.

As screening guidelines continue to be refined and revised in coming years, historical effects of prior recommendations should be considered, as should the ways in which these policies affect particular racial and ethnic groups.

## Supplementary Material

pkae106_Supplementary_Data

## Data Availability

The case data analyzed in this study are available from the SEER website at https://seer.cancer.gov/data-software. The code analyzing these data is publicly available at https://github.com/FredHutch/PCa-PSA-Age-Trends.
