# Supplementary material for: Trends in age and prostate-specific antigen at prostate cancer diagnosis between 2010 and 2019
Source: JNCI Cancer Spectr. 2024 Oct 23;8(6):pkae106. doi: 10.1093/jncics/pkae106 (PMC11578289; doi:10.1093/jncics/pkae106)
Supplement: pkae106_Supplementary_Data [file pkae106_supplementary_data.pdf]

Supplementary Materials for "Trends in Age and Prostate-Specific Antigen at Prostate Cancer Diagnosis between 2010 and 2019"

Lukas Owens, Ojas Brahme, Roman Gulati, Ruth Etzioni

Division of Public Health Sciences, Fred Hutchinson Cancer Center, Seattle, WA, USA

Supplementary Table 1

Quantile regression of age at diagnosis on (A) year of diagnosis and (B) year of diagnosis and race and ethnic origin in Surveillance, Epidemiology and End Results database from 2010 to 2019

	**25th percentile**			**50th percentile**			**75th percentile**			**85th percentile**		
	Coefficient	95% CI	*p*-value	Coefficient	95% CI	*p*-value	Coefficient	95% CI	*p*-value	Coefficient	95% CI	*p*-value
**(A) Year Only**												
Intercept	56.00	55.92, 56.08	<0.001	61.86	61.82, 61.89	<0.001	67.00	66.97, 67.03	<0.001	70.00	69.82, 70.18	<0.001
Year	0.25	0.24, 0.26	<0.001	0.14	0.13, 0.15	<0.001	0.14	0.13, 0.15	<0.001	0.14	0.11, 0.17	<0.001
**(B) Year and Race**												
Intercept	56.75	56.66, 56.84	<0.001	62.20	62.11, 62.29	<0.001	67.40	67.31, 67.49	<0.001	70.71	70.58, 70.85	<0.001
Year	0.25	0.23, 0.27	<0.001	0.20	0.18, 0.22	<0.001	0.20	0.19, 0.21	<0.001	0.14	0.12, 0.16	<0.001
Race												
NH White	–	–	–	–	–	–	–	–	–	–	–	–
NH Black	-2.75	-2.96, -2.54	<0.001	-2.53	-2.72, -2.34	<0.001	-2.40	-2.54, -2.26	<0.001	-2.71	-2.85, -2.58	<0.001
Hispanic	-1.08	-1.34, -0.83	<0.001	-0.20	-0.47, 0.07	0.146	-0.40	-0.58, -0.22	<0.001	-0.71	-0.85, -0.58	<0.001
NH API	1.25	0.97, 1.53	<0.001	0.80	0.45, 1.15	<0.001	1.60	1.18, 2.02	<0.001	1.29	1.00, 1.57	<0.001
NH AI/AN	0.12	-2.11, 2.36	0.913	0.80	0.71, 0.89	<0.001	-0.40	-2.63, 1.83	0.725	-0.71	-3.39, 1.96	0.601
Year : Race												
NH White	–	–	–	–	–	–	–	–	–	–	–	–
NH Black	0.00	-0.03, 0.03	>0.999	-0.03	-0.06, 0.00	0.033	-0.08	-0.10, -0.05	<0.001	-0.14	-0.18, -0.11	<0.001
Hispanic	-0.08	-0.12, -0.05	<0.001	-0.20	-0.24, -0.16	<0.001	-0.20	-0.25, -0.15	<0.001	-0.14	-0.19, -0.10	<0.001
NH API	-0.08	-0.14, -0.02	0.007	0.02	-0.04, 0.09	0.492	-0.20	-0.30, -0.10	<0.001	-0.14	-0.23, -0.06	0.001
NH AI/AN	-0.12	-0.53, 0.28	0.548	-0.20	-0.22, -0.18	<0.001	-0.09	-0.50, 0.32	0.669	-0.03	-0.52, 0.46	0.899

CI: confidence interval; NH: Non-Hispanic; API: Asian/Pacific Islander; AI/AN: American Indian/Alaska Native

Supplementary Table 2

Quantile regression of PSA at diagnosis on (A) year of diagnosis and (B) year of diagnosis and race and ethnic origin in Surveillance, Epidemiology and End Results database from 2010 to 2019

	**25th percentile**			**50th percentile**			**75th percentile**			**85th percentile**		
	Coefficient	95% CI	*p*-value	Coefficient	95% CI	*p*-value	Coefficient	95% CI	*p*-value	Coefficient	95% CI	*p*-value
**(A) Year Only**												
Intercept	4.55	4.54, 4.56	<0.001	6.01	5.99, 6.04	<0.001	9.40	9.33, 9.47	<0.001	13.60	13.42, 13.78	<0.001
Year	0.08	0.07, 0.08	<0.001	0.14	0.14, 0.15	<0.001	0.34	0.32, 0.35	<0.001	0.80	0.75, 0.85	<0.001
**(B) Year and Race**												
Intercept	4.42	4.41, 4.44	<0.001	5.83	5.81, 5.86	<0.001	8.70	8.63, 8.77	<0.001	12.00	11.83, 12.17	<0.001
Year	0.08	0.07, 0.08	<0.001	0.13	0.13, 0.14	<0.001	0.30	0.28, 0.32	<0.001	0.65	0.61, 0.69	<0.001
Race												
NH White	–	–	–	–	–	–	–	–	–	–	–	–
NH Black	0.38	0.33, 0.42	<0.001	0.82	0.73, 0.92	<0.001	2.90	2.61, 3.19	<0.001	7.20	6.30, 8.10	<0.001
Hispanic	0.33	0.28, 0.38	<0.001	0.62	0.53, 0.72	<0.001	1.71	1.42, 2.01	<0.001	3.82	2.95, 4.70	<0.001
NH API	0.58	0.52, 0.63	<0.001	1.04	0.91, 1.18	<0.001	2.30	1.86, 2.74	<0.001	4.50	3.55, 5.45	<0.001
NH AI/AN	0.87	0.54, 1.21	<0.001	2.09	1.41, 2.78	<0.001	7.50	3.55, 11.45	<0.001	29.90	9.19, 50.61	0.005
Year : Race												
NH White	–	–	–	–	–	–	–	–	–	–	–	–
NH Black	0.00	-0.01, 0.01	>0.999	0.01	-0.01, 0.03	0.299	0.13	0.06, 0.19	<0.001	0.46	0.25, 0.67	<0.001
Hispanic	0.00	-0.01, 0.01	0.492	0.01	-0.01, 0.03	0.341	0.19	0.11, 0.26	<0.001	0.73	0.49, 0.96	<0.001
NH API	0.00	-0.01, 0.01	>0.999	-0.01	-0.04, 0.02	0.542	0.05	-0.04, 0.14	0.268	0.15	-0.10, 0.40	0.244
NH AI/AN	0.00	-0.07, 0.07	>0.999	-0.06	-0.19, 0.07	0.381	0.10	-0.55, 0.75	0.763	-0.95	-4.30, 2.40	0.578

PSA: Prostate-specific antigen; CI: confidence interval; NH: Non-Hispanic; API: Asian/Pacific Islander; AI/AN: American Indian/Alaska Native
